# Salivary Glycopatterns as Potential Non-Invasive Biomarkers for Diagnosing and Reflecting Severity and Prognosis of Diabetic Nephropathy

**DOI:** 10.3389/fendo.2022.790586

**Published:** 2022-03-31

**Authors:** Qiuxia Han, Xiaochen Wang, Xiaonan Ding, Jing Hao, Qi Li, Jifeng Wang, Hanjie Yu, Zhen Tang, Fuquan Yang, Guangyan Cai, Dong Zhang, Hanyu Zhu

**Affiliations:** ^1^ Department of Nephrology, The First Medical Centre, Chinese PLA General Hospital, Chinese PLA Institute of Nephrology, State Key Laboratory of Kidney Diseases, National Clinical Research Center of Kidney Diseases, Beijing Key Laboratory of Kidney Disease, Beijing, China; ^2^ School of Medicine, Nankai University, Tianjin, China; ^3^ The Key Laboratory of Protein and Peptide Pharmaceuticals, Laboratory of Proteomics, Institute of Biophysics, Chinese Academy of Sciences, Beijing, China; ^4^ Laboratory for Functional Glycomics, College of Life Sciences, Northwest University, Xi’an, China

**Keywords:** saliva, glycopatterns, diagnosis, prognosis, diabetic nephropathy, non-invasive biomarkers

## Abstract

Discriminating between diabetic nephropathy (DN) and non-diabetic renal disease (NDRD) can help provide more specific treatments. However, there are no ideal biomarkers for their differentiation. Thus, the aim of this study was to identify biomarkers for diagnosing and predicting the progression of DN by investigating different salivary glycopatterns. Lectin microarrays were used to screen different glycopatterns in patients with DN or NDRD. The results were validated by lectin blotting. Logistic regression and artificial neural network analyses were used to construct diagnostic models and were validated in in another cohort. Pearson’s correlation analysis, Cox regression, and Kaplan–Meier survival curves were used to analyse the correlation between lectins, and disease severity and progression. Liquid chromatography–tandem mass spectrometry (LC-MS/MS) and bioinformatics analyses were used to identify corresponding glycoproteins and predict their function. Both the logistic regression model and the artificial neural network model achieved high diagnostic accuracy. The levels of *Aleuria aurantia* lectin (AAL), *Lycopersicon esculentum* lectin (LEL), *Lens culinaris* lectin (LCA), *Vicia villosa* lectin (VVA), and *Narcissus pseudonarcissus* lectin (NPA) were significantly correlated with the clinical and pathological parameters related to DN severity. A high level of LCA and a low level of LEL were associated with a higher risk of progression to end-stage renal disease. Glycopatterns in the saliva could be a non-invasive tool for distinguishing between DN and NDRD. The AAL, LEL, LCA, VVA, and NPA levels could reflect the severity of DN, and the LEL and LCA levels could indicate the prognosis of DN.

## Introduction

Diabetes will be the seventh leading cause of mortality by 2030, and the number of diabetes patients is expected to exceed 693 million by 2045 (1, 2). Diabetic nephropathy (DN), a serious complication of diabetes, occurs in 20% to 40% of patients with type 2 diabetes mellitus (T2DM) and is a huge economic burden on our society (3). Many scholars have found that some patients with both diabetes and kidney disease have different clinical manifestations and treatment sensitivity from typical DN patients, and this disease was coined as non-diabetic renal disease (NDRD) (4). After a kidney biopsy in patients with both diabetes and kidney disease, nearly two-thirds of patients were diagnosed with NDRD (5–7). The main types of NDRD are diabetes combined membranous nephropathy (MN), immunoglobulin A nephropathy (IgAN), and focal segmental glomerulosclerosis (FSGS) (8). DN and NDRD differ in many aspects such as pathological characteristics, clinical manifestations, treatment response, disease progression and prognosis (9). Patients with NDRD were found to have a better prognosis than patients with DN if they could receive timely treatment (10). Therefore, it is important to accurately diagnose DN and NDRD. Some doctors distinguish between DN and NDRD based on clinical experience, which may be inaccurate, leading to the risk of delaying the timing of treatment. The gold standard for the diagnosis of DN and NDRD is percutaneous renal puncture, which is a time-consuming, invasive, and expensive procedure (5). Therefore, it is of great clinical value to find a convenient and non-invasive method for differentiating DN from NDRD.

Currently, saliva is recognised as a convenient way to assess human pathological conditions, owing to its advantages in collection and storage (11, 12). Saliva is a complex oral secretion originating from the salivary gland, which is composed of many secreted proteins, electrolytes, and other substances (11). Saliva is an ideal biological fluid, which contains various substances that can reflect the health of the body (11). Salivary proteins are also widely used in the diagnosis of various diseases, such as Sjogren’s syndrome, cystic fibroma, and cancer (13–16).

Lectin is a glycan-binding protein synthesised and secreted by both animal cells and plant cells. Lectin can distinguish glycopatterns according to slight structural differences and can combine with the sugar chain structure on a specific glycolipid or glycoprotein to form a covalent bond (17). Compared with antibodies, lectin costs less, is easy to obtain, and has a higher specific affinity for some glycosyl groups, which can aid in-depth analysis (18, 19). The techniques that can be used for the detection of glycoproteins include Western blotting, mass spectrometry (MS) analysis, and chromatography (20). However, these techniques have several shortcomings, such as time consumption and low efficiency, which can be circumvented using the lectin microarray technique (17). The present study used the emerging high-throughput glycosylation technology, which enabled lectin microarrays to study samples such as serum, urine, and saliva glycosylation while observing a variety of different binding reactions.

Protein glycosylation is an important and abundant post-translational modification (21). It is a process in which saccharides are transferred to the polypeptide chain skeleton, mediated by glycosyltransferase, glycosidase, and other enzymes (22). Glycosylation mainly occurs in the endoplasmic reticulum and Golgi apparatus of cells (22), and glycosylated proteins play an important role in cellular activities (22, 23).

There are two main types of glycosylated proteins in mammals: *O*-glycosylated proteins (such as mucins) and *N*-glycosylated proteins (such as erythropoietins) (24–26). Glycosylation plays an important role in the folding and conformation of stable proteins and assists a variety of biological processes through cell adhesion and recognition (27). Abnormal glycosylation is associated with many diseases, such as tumours, inflammation, and neurodegenerative diseases (28, 29). This study aimed to provide a non-invasive diagnostic tool to distinguish between DN and NDRD by analysing salivary glycopatterns and to identify biomarkers that reflect the severity and prognosis of DN.

## Methods

### Recruitment Cohort

Human whole saliva was obtained from the Chinese PLA General Hospital. The study was approved by the Ethics Committee of the Chinese PLA General Hospital (No. S2014-012-01). Participants signed a written informed consent form upon collection of their saliva. This study was conducted in accordance with the Declaration of Helsinki.

**Figure 1:**
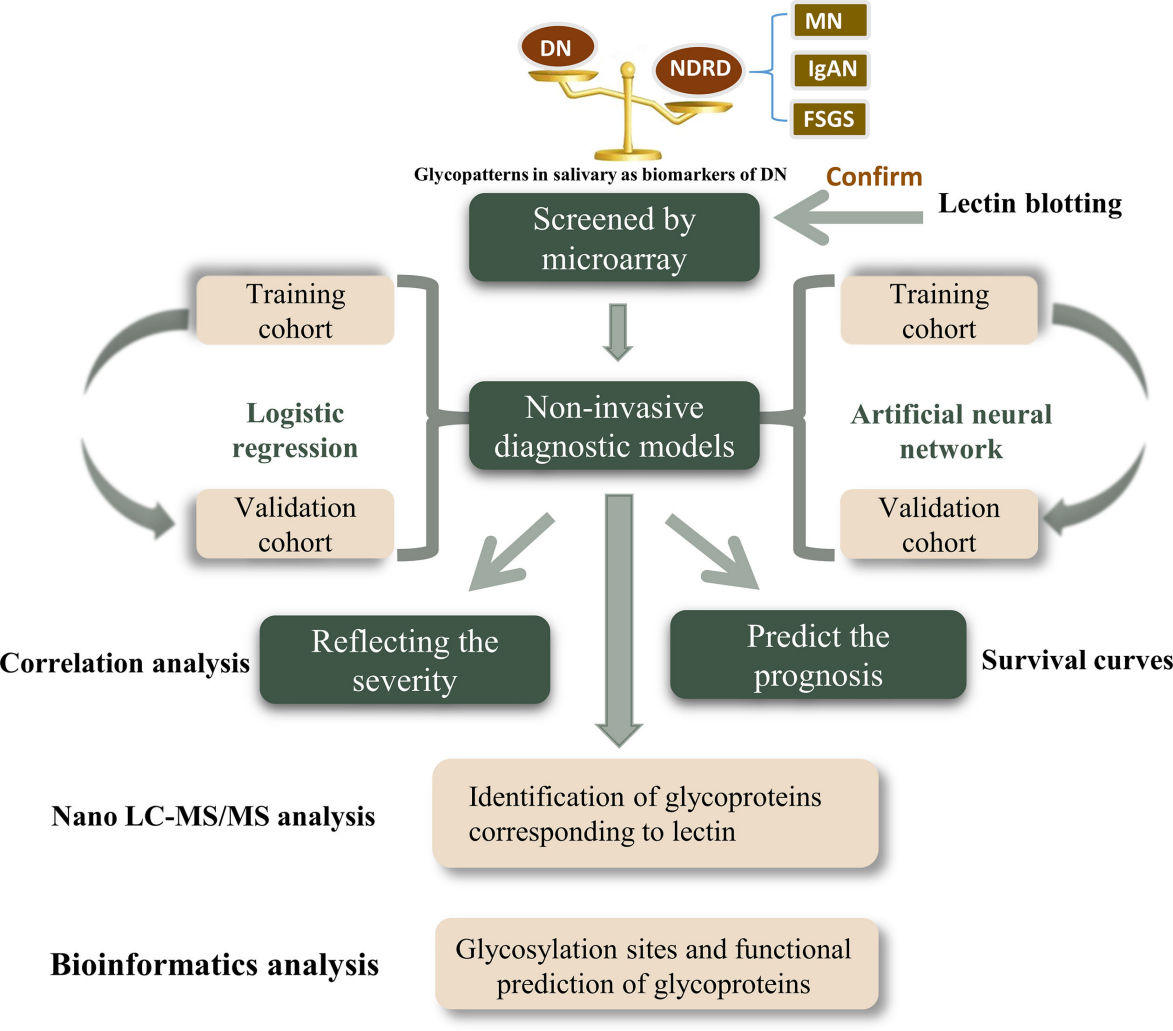
Schematic illustration of the present research. DN, diabetic nephropathy; NDRD, non-diabetic renal disease; MN, membranous nephropathy; IgAN, immunoglobulin A nephropathy; FSGS, focal segmental glomerulosclerosis.

Between January 2016 and October 2020, 181 eligible subjects were enrolled in this study, and the saliva of each patient was individually tested using lectin microarrays. **Figure 1** shows the design of the study. **Table S1** summarises the basic clinical characteristics of the training cohort and validation cohort. **Table S2** shows the clinical information related to diabetes. With the use of a confidence level of 0.95, a power of 0.8, a distance from mean to limits of 0.3, an SD of 0.4, and a two-sided interval, the required sample size was calculated to be 17.

The inclusion criteria were as follows: diagnosis of type 2 diabetes; pathological diagnosis of DN or NDRD; age of over 18 years; renal puncture biopsy that was performed in our hospital; and agreement to participate in the study after signing the voluntary informed consent form. The exclusion criteria were as follows: incomplete medical history; presence of other types of secondary renal disease such as lupus nephritis and Henoch–Schönlein purpura nephritis; patients with hereditary kidney disease; and combined urinary tract infection, malignant tumour, or pregnancy. All patients with DN and NDRD were diagnosed with pathological diagnosis through renal biopsy. The DN pathological stages were classified according to the Renal Pathology Society classification system (30). The diagnosis of NDRD followed the 2007 Kidney Disease Outcomes Quality Initiative guidelines (31). The diagnosis of the pathology was independently reviewed by two qualified pathologists.

### Whole Saliva Collection

The collection methods were based on previous studies (32–34). In short, between 9 a.m. and 11 a.m., unstimulated saliva was collected at least 3 h after the last meal. Saliva samples were collected immediately after oral rinsing with sterile saline, placed on ice, and centrifuged at 10,000 × *g* at 4°C for 15 min to remove insoluble precipitates. The supernatant (1 ml) was transferred to a new tube, and 10 μl of protease cocktail inhibitor (1:100 [v/v], Sigma-Aldrich, St. Louis, MO, USA) was added. Bicinchoninic acid (BCA) assays (Beyotime Biotechnology, Shanghai, China) were used to measure the protein concentration of each saliva sample in triplicate. The treated saliva specimens were stored at −80°C until use.

### Lectin Microarrays

A lectin microarray was obtained by synthesising 37 lectins with different binding preferences for the N and O chains (32). Salivary proteins were labelled with Cy3 dye (GE Healthcare, Boston, MA, USA). Cy3-labelled salivary proteins measuring 4 μg was mixed with 120 μg of incubation buffer and applied to lectin microarrays for 3 h at 37°C. Only sugar chains with a specific structure can bind to the corresponding lectin. Therefore, the fluorescence intensity of lectin represents the expression level of the corresponding glycoprotein.

### Lectin Blotting

Lectin blotting was used to analyse the expression levels of the polysaccharides. Each group of concentrated salivary proteins was transferred to a polyvinylidene fluoride membrane after 10% sodium dodecyl sulphate (SDS)–polyacrylamide gel electrophoresis (PAGE). The membrane was then incubated with either Cy5-labelled LacNAc and poly-LacNAc conjugated with *Lycopersicon esculentum* lectin (LEL) or GalNAC terminus, GalnacαSer/Thr(TN), and GalNAcα1-3Gal conjugated with VVA.

### Artificial Neural Network Prediction

An artificial neural network is a generalised model of neurobiological systems (35). Essentially, it is an attempt to simulate the human brain. Artificial neural networks can learn and replicate complex or non-linear input–output relationships by using simulated neurons. In this study, the NeuralNet Package in R (https://CRAN.R-roject.org/package=neuralnet) was used for the artificial neural network analysis. Default parameters were used, except that the argument of the hidden was fitted as H = C (30, 0).

### Isolation of Glycoproteins by *Lycopersicon esculentum* Lectin-Coupled Magnetic Particle Conjugate

The proteins identified by LEL were isolated as previously described (36). Briefly, after dissolving 400 μg of LEL in 400 μl of binding solution (0.1 M of Tris-HCl, 150 mM of NaCl, 1 mM of CaCl_2_, 1 mM of MgCl_2_, and 1 mM of MnCl_2_, pH 7.4), epoxysilane-coated magnetic particles (homemade) were added and incubated in binding buffer for 3 h. The 1× carbo-free blocking solution (Vector Labs, Burlingame, CA, USA) was used to block the LEL-coupled magnetic particle conjugate at room temperature for 1 h after washing three times with washing buffer (binding solution containing 0.02% Tween-20 (v/v)). Next, 1 mg of salivary protein was added to the conjugate, and the mixture was shaken and incubated to enrich glycoproteins for 3 h at room temperature. The solution was then washed thrice in washing buffer to remove non-specifically bound proteins, and the specific glycoproteins were eluted using the competitive elution buffer (100 mM of lactose). The BCA protein assay kit was used to determine the concentration of the isolated glycoproteins in triplicate.

### Nano Liquid Chromatography–Tandem Mass Spectrometry Analysis

The Orbitrap Exploris 480 (Thermo Scientific, Waltham, MA, USA) equipped with an Easy n-LC 1200 HPLC system (Thermo Scientific) was used to perform all nanoscale liquid chromatography–tandem MS (LC-MS/MS) experiments. A 100 μm id × 2 cm fused silica trap column filled with reversed-phase silica gel (Reprosil-Pur C18 AQ, 5 μm, Dr Maisch GmbH, Ammerbuch, Germany) was used to load the peptides, and a 75 μm id × 20 cm C18 column filled with reversed-phase silica gel (Reprosil-Pur C18 AQ, 3 μm, Dr Maisch GmbH) was used for separation. The peptides bound to the column were eluted with a linear gradient for 73 min. Formic acid (FA; 0.1%) in water formed solvent A, and 80% acetonitrile and 0.1% FA formed solvent B. The segmented gradient was 4%–9% B, 3 min; 9%–20% B, 22 min; 20%–30% B, 20 min; 30%–40% B, 15 min; 40%–95% B, 3 min; and 95% B, 10 min, at a flow rate of 300 nl/min. Data-related acquisition mode was used to acquire MS data at a high resolution of 60,000 (*m*/*z* 200) within a mass range of 350–1,500 *m*/*z*. The target value was 3.00E+06, and the maximum injection time was 22 ms. The data-related mode was selected as the cycle time mode and set to 2 s. Precursor ions were selected from each full MS scan with an isolation width of 1.6 *m*/*z* for fragmentation in the Ion Routing Multipole with a normalised collision energy of 28%. MS/MS spectra were collected with a resolution of 15,000 at *m*/*z* 200. The target value was 7.50E+04, and the maximum injection time was 22 ms. The dynamic exclusion time was 40 s. For nano electrospray ion source setting, the spray voltage was 2.0 kV, with no sheath gas flow, and the capillary temperature was 320°C.

### Database Searching and Analysis

The Proteome Discovery version 2.4.1.15 with Sequest HT search engine was used to analyse the raw LS-MS/MS data and identify the corresponding proteins. Data from saliva samples were used to search the UniProt human protein database (updated on September 2018). The proteolytic enzyme used was trypsin, and two missed cleavages were allowed. The tolerance level of the precursor was 10 ppm for MS, and the product ion tolerance was 0.02 Da for MS. Carbamidomethylation of cysteines was set as the fixed modification, and methionine oxidation was set as the variable modification. False discovery rate (FDR) analysis was performed with Percolator with the setting of FDR < 1% for protein identification. The areas of identified peptides were used for label-free protein quantification on Proteome Discovery. Only unique and razor peptides of proteins were selected for relative quantification. Normalisation mode was selected as the total peptide amount to correct for experimental bias.

### Bioinformatics Analysis

The biological function and significance of proteins were obtained using Gene Ontology (GO) analysis. Blast2GO software (version 6.0) was used to characterise the biological process, molecular function, and cellular component of each protein. In addition, DAVID Bioinformatics Resources (version 6.8) was used to analyse the pathway enrichment of differential proteins. Kyoto Encyclopedia of Genes and Genomes (KEGG) pathway enrichment analysis was performed by mapping the thresholds of the background signal in the human genome with a count > 4 and a p-value <0.05. The STRING database was used to perform functional interaction network analysis of differential proteins. Proteins with an interaction score of 0.4 and interactions derived from text-mining were excluded.

### Statistical Analysis

To reduce possible systematic variations, raw data from the lectin microarray were normalised. The median of the effective data points of each lectin was globally normalised to the sum of the median of all the effective data points in a block, which is called the normalised fluorescence intensity (NFI). The normalised data were further analysed by hierarchical cluster analysis (HCA) using Expander 6.0 (http://acgt.cs.tau.ac.il/expander/), and principal component analysis (PCA) was performed using the Multi-Variate Statistical Package (Vision 3.1 Kovach Computing Services, Wales, UK).

Normal distribution data were expressed as mean ± SD and compared using unpaired Student’s t-test. Abnormal distribution data were represented as medians of the corresponding 25th and 75th percentiles (quaternary ranges) and compared using the Mann–Whitney U test. The DN diagnosis model was established according to the abundance of glycopatterns using logistic regression analysis. A receiver operating characteristic (ROC) curve was used to evaluate the diagnostic performance. Pearson’s correlation was used to evaluate the correlation between lectin levels and clinical and pathological parameters related to the severity of DN. To analyse the prognosis of the DN patients, the data based on the lectin levels of patients and time to enter the dialysis phase were obtained using Cox regression, Kaplan–Meier survival curves, and the log-rank test. Differences were considered statistically significant at *p* < 0.05. Statistical analyses were performed using SPSS Statistics 21.0 software (version 21.0, SPSS, Chicago, IL, USA) and GraphPad Prism software (version 8, San Diego, CA, USA).

## Results

### Alterations of Glycopatterns Between Diabetic Nephropathy and Non-Diabetic Renal Disease

**Figure 2:**
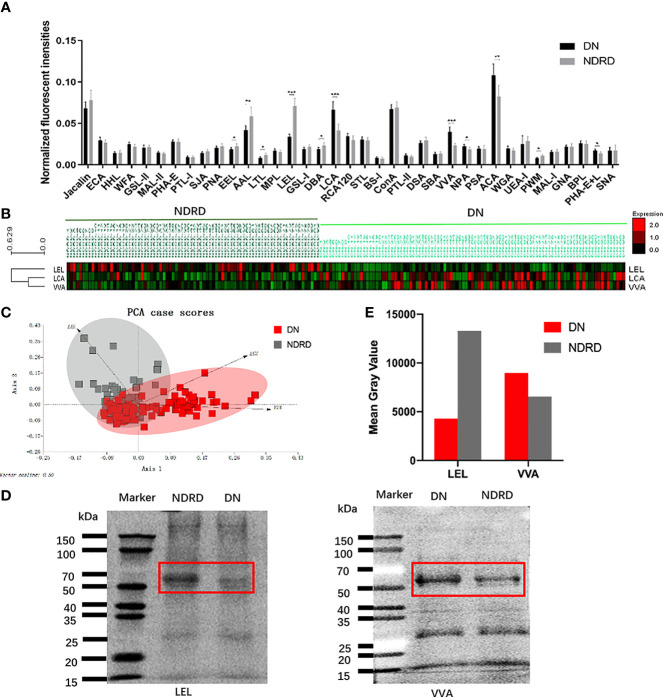
Exhibition and confirmation of differentiation with DN and NDRD using lectins. **(A)** Comparison of all candidate lectins in salivary samples from DN and NDRD patients. The normalised fluorescence intensities of 37 lectins from DN and NDRD patients were compared based on fold-change and one-way ANOVA (*p < 0.05, **p < 0.01, and ***p < 0.001). Data are presented as the mean ± 95% CI. **(B)** Hierarchical clustering analysis of the three lectins with significant differentiation of NFIs between DN and NDRD. Glycan profiles of DN and NDRD patients were clustered (average linkage, correlation similarity). Samples are listed in columns, and lectins are listed in rows. The colour intensity of each square indicates the expression levels relative to other data. Red, high; green, low; black, medium. **(C)** The normalised glycopattern abundances of three lectins related to the two groups were subjected to principal component analysis (PCA). DN and NDRD samples were visualised by red and grey shadows, respectively. **(D)** Confirmation of salivary glycopatterns from DN and NDRD groups using LEL and VVA lectins was performed by lectin blotting. **(E)** Mean grey value of each apparent difference band was obtained using ImageJ. DN, diabetic nephropathy; NDRD, non-diabetic renal disease; NFIs, normalised fluorescence intensities; LEL, *Lycopersicon esculentum* lectin; VVA, *Vicia villosa* lectin.

The NFIs for each lectin were summarised as the mean ± 95% CI (**Figure 2A**). The NFIs of each lectin from DN and NDRD were compared, and the results showed that the NFIs of *Lens culinaris* lectin (LCA) (*p* < 0.001), *Vicia villosa* lectin (VVA) (*p* < 0.001), *Narcissus pseudonarcissus* lectin (NPA) (*p* < 0.05), *Amaranthus caudatus* lectin (ACA) (*p* < 0.01), and *Phaseolus vulgaris* lectin (PHA-E+L) (*p* < 0.05) were significantly higher in the DN group than in the NDRD group, whereas *Euonymus europaeus* lectin (EEL) (*p* < 0.05), *Aleuria aurantia* lectin (AAL) (*p* < 0.01), *Lotus tetragonolobus* lectin (LTL) (*p* < 0.05), LEL (*p* < 0.001), *Dolichos biflorus agglutinin* lectin (DBA) (*p* < 0.05), and *Phytolacca americana* lectin (PWM) (*p* < 0.05) showed lower binding signals in the DN group than in the NDRD group. One category included the cluster of LCA and VVA, whereas the other category contained a cluster of LEL (**Figure 2B**). The LEL, VVA, and LCA with the highest significance were selected to show the recognition power for all saliva samples, depending on the results of the NFIs. Despite the small overlapping area, the LEL, VVA, and LCA could separate patients with DN from those with NDRD using PCA, as shown in **Figure 2C**. According to the results of the lectin microarray, the LEL and VVA were selected to perform lectin blotting to confirm the abundance of glycopatterns between the DN and NDRD patients. The red frames highlight the protein bands that showed differences between the DN and NDRD groups. The lectin blotting results show that LEL lectin specifically bound glycoproteins with a molecular weight of about 70 kDa, and their expression in patients with DN was lower than that in patients with NDRD. In contrast, VVA lectin is specifically bound to glycoproteins with a molecular weight of about 60 kDa, and their expression in patients with DN was higher than that in patients with NDRD (**Figures 2D, E**).

### Establishment and Verification of the Diagnostic Model

A total of 181 participants were enrolled in this study. The baseline characteristics of the subjects are presented in **Table S1**. There were no statistical differences in the information between the training and validation cohorts, which indicates that the model is not biased. Logistic regression and artificial neural network analysis were applied to establish two diagnostic models for DN and NDRD, depending on the data of all candidate lectins in the training cohort. Diabetes-related clinical information of the DN and NDRD groups is presented in **Table S2**.

**Figure 3:**
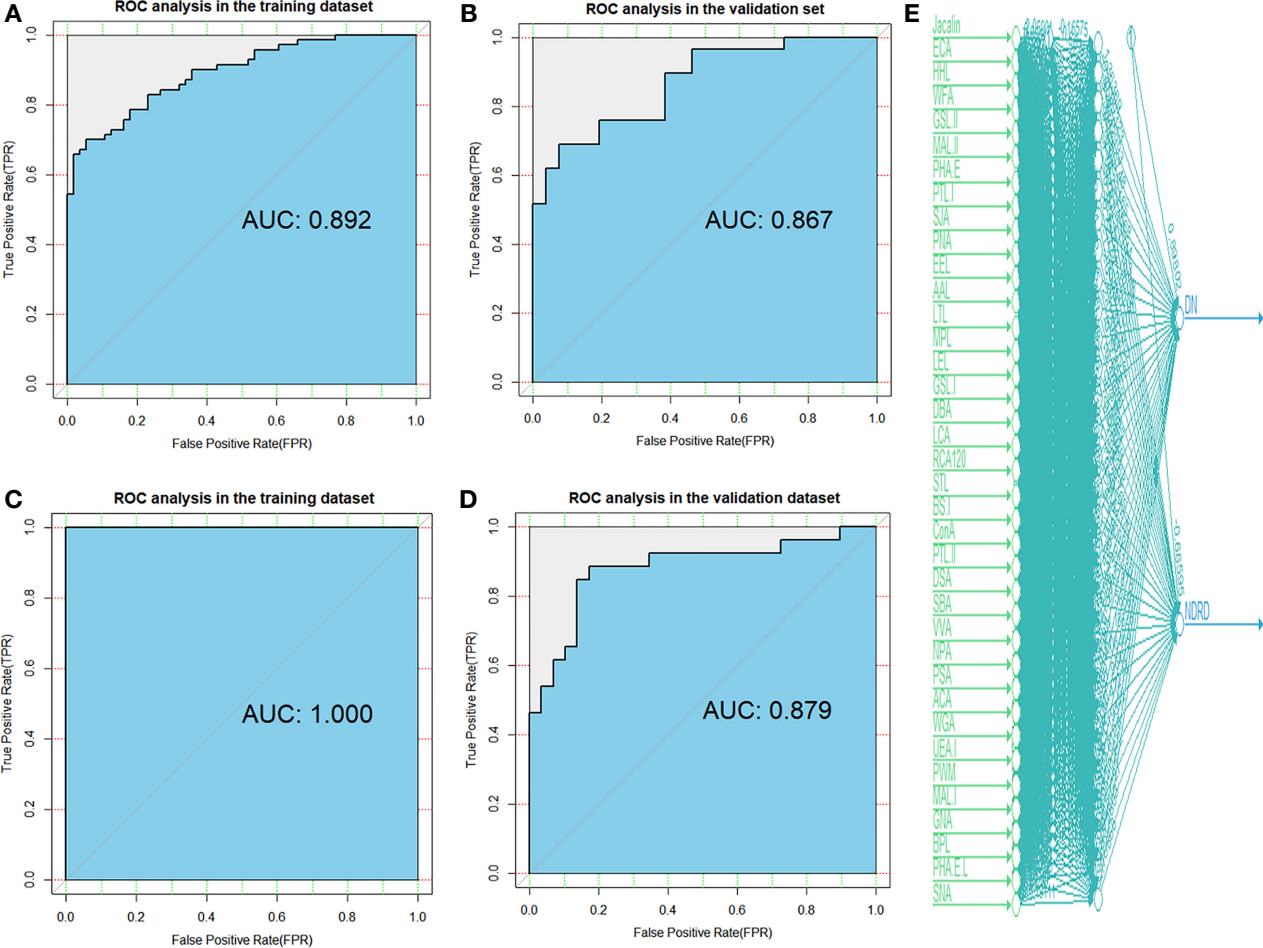
Diagnostic accuracy of selected lectins and models was determined by ROC analysis with logistic regression and artificial neural network methods. ROC analysis for models constructed by logistic regression in the training **(A)** and validation **(B)** cohorts. ROC analysis for models constructed by artificial neural network in the training **(C)** and validation **(D)** cohorts. **(E)** A total of 37 candidate lectins for all patients are displayed in artificial neural network analysis. DN, diabetic nephropathy; NDRD, non-diabetic renal disease; ROC, receiver operating characteristic.

In addition, the ROC method was used to test the diagnostic models in the training and validation cohorts using the logistic regression (**Figures 3A, B**) and artificial neural network (**Figures 3C, D**) methods. We used the neural network algorithm to construct a binomial diagnosis model with 37 lectins as feature variables, and the schematic diagram of its construction process is shown in **Figure 3E**. The area under the curve (AUC) of the logistic regression model in the training and validation cohorts was 0.892 and 0.867, respectively, and the AUCs of the artificial neural network analysis model were 1.000 and 0.879, respectively. Our results showed that the models we developed in the training cohorts also performed very well in the validation cohorts, indicating that the non-invasive diagnostic model we established has good applicability and is reliable. The parameters of the evaluation model are presented in **Table S3**.

The diagnostic model of the logistic regression is shown in Eq. 1:


(1) $$Model\;DN=\frac{1}{1+e^{-(2.0579-74.1810*\text{LEL}+15.3656*\text{VVA}+23.3397*\text{LCA}+1.5249*\text{BPL})}}$$

### Association of Glycopatterns and Severity of Diabetic Nephropathy Patients

To investigate the correlation between glycopatterns and the severity of DN, we analysed the expression levels of lectin and the clinical and pathological parameters associated with DN severity using Pearson’s correlation analysis (**Table 1**). The LEL levels correlated positively with the estimated glomerular filtration rate (eGFR) (*p* < 0.001) but were negatively correlated with the blood urea nitrogen (BUN), serum creatinine (SCr), classes of glomerular lesions, and scores of interstitial and vascular lesions (all *p* < 0.001). The LCA level correlated negatively with the eGFR (*p* < 0.001) only but correlated positively with the BUN, SCr, classes of glomerular lesions, and scores of interstitial and vascular lesions (all *p* < 0.001). The VVA level correlated negatively with the eGFR (*p* < 0.001) only but correlated positively with proteinuria (*p* < 0.001), SCr (*p* < 0.001), classes of glomerular lesions (*p* < 0.01), and scores of interstitial and vascular lesions (*p* < 0.001). The AAL and NPA levels were negatively correlated with the BUN and classes of glomerular lesions (all *p* < 0.05), respectively, whereas there was no significant correlation between the EEL, LTL, DBA, ACA, PWM, BPL, and PHA-E+L levels and the clinical and pathological parameters associated with DN severity.

**Table 1:** Pearson’s correlation of expression levels of glycopatterns in the saliva and clinical and pathological indicators related to the severity of diabetic nephropathy.

	Proteinuria (g/24 h)	BUN (mmol/L)	eGFR (ml/min/1.73 m^2^)	Scr (μmol/L)	Classes of glomerular lesions	Scores of interstitial and vascular lesions
**EEL**	0.049	−0.040	−0.179	0.111	0.066	0.160
**AAL**	−0.119	−0.215*	0.102	−0.055	0.005	−0.084
**LTL**	0.130	−0.085	−0.045	0.095	0.036	0.097
**LEL**	−0.084	−0.350***	0.884***	−0.768***	−0.739***	−0.769***
**DBA**	0.027	−0.038	0.009	−0.004	−0.023	0.010
**LCA**	0.043	0.402***	−0.757***	0.783***	0.573***	0.769***
**VVA**	0.606***	−0.013	−0.377***	0.450***	0.276**	0.486***
**NPA**	−0.038	−0.084	0.192	−0.163	−0.206*	−0.167
**ACA**	−0.025	−0.165	0.097	−0.030	0.023	−0.084
**PWM**	−0.056	0.077	0.092	−0.113	0.039	−0.138
**PHA-E+L**	−0.025	0.057	−0.044	0.040	0.102	0.011

The estimated glomerular filtration rate (eGFR) was calculated by the Chronic Kidney Disease Epidemiology Collaboration (CKD-EPI) equation.

BUN, blood urea nitrogen; Scr, serum creatinine; EEL, Euonymus europaeus lectin; AAL, Aleuria aurantia lectin; LTL, Lotus tetragonolobus lectin; LEL, Lycopersicon esculentum lectin; DBA, Dolichos biflorus agglutinin lectin; LCA, Lens culinaris lectin; VVA, Vicia villosa lectin; NPA, Narcissus pseudonarcissus lectin; ACA, Amaranthus caudatus lectin; PWM, Phytolacca americana lectin.

*p < 0.05; **p < 0.01; ***p < 0.001.

### Association of Glycopatterns and Prognosis of Diabetic Nephropathy Patients

**Figure 4:**
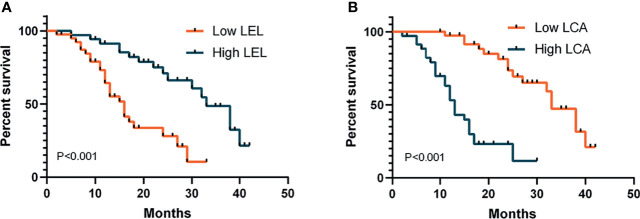
Kaplan–Meier analysis of dialysis-free survival in patients with diabetic nephropathy. Subjects were dichotomised based on the mean of the covariates: **(A)** 0.036 for LEL; **(B)** 0.060 for LCA. p-Values refer to log-rank tests. LEL, *Lycopersicon esculentum* lectin; LCA, *Lens culinaris* lectin.

To investigate the association between glycopatterns and dialysis-free survival in patients with DN, multivariate Cox regression analysis was performed (**Table S4**). Higher LEL levels were associated with a reduced risk of developing end-stage renal disease (ESRD) and receiving dialysis therapy [*p* < 0.001, hazard ratio (HR) < 0.001]. However, higher LCA levels increased the risk of progressing to ESRD and receiving dialysis therapy (*p* < 0.01, HR = 730,046.848). The levels of AAL, VVA, and NPA were not significantly associated with DN progression. The Kaplan–Meier analysis was used to further investigate the relationship between the LEL and VVA levels and the dialysis-free survival of DN patients (**Figure 4**). The subjects were dichotomised based on the mean of the covariates (0.036 for LEL and 0.060 for LCA). Prolonged time to progression and dialysis was exhibited in DN patients with a high level of LEL, whereas a shorter time to progression and dialysis was observed in patients with a high level of LCA.

### Characterisation of Protein by Liquid Chromatography–Tandem Mass Spectrometry

**Figure 5:**
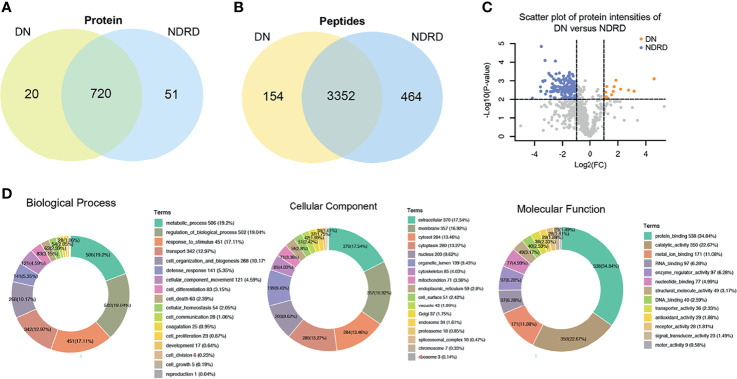
Bioinformatics analysis of isolated glycoproteins from DN and NDRD. **(A)** Venn diagram of isolated proteins from DN and NDRD using LEL-coupled magnetic particle conjugates. **(B)** Venn diagram of isolated peptides from DN and NDRD using LEL-coupled magnetic particle conjugates. **(C)** Scatter plot of protein levels between DN and NDRD. y-Axis correspond to p-values (−log10) versus protein log2 fold-change (x-axis) in DN/NDRD. Colour indicates upregulation (orange) (fold-change > 2, p < 0.01) and downregulation (blue) (fold-change < 0.5, p < 0.01). Black represents the level of proteins without statistically significant difference between NDRD and DN. **(D)** Blast2GO was used to classify identified proteins into biological process, cellular component, and molecular function. DN, diabetic nephropathy; NDRD, non-diabetic renal disease; LEL, *Lycopersicon esculentum* lectin.

Based on the above results, we found that the glycoproteins that specifically bind to LEL lectin are suitable for the non-invasive diagnosis of DN. They also reflect the severity and prognosis of the disease. Therefore, we further studied the relationship between these glycoproteins and the biological processes related to DN. The proteins from DN and NDRD were respectively isolated and characterised using lectin affinity separation and LC-MS/MS. A total of 3,506 (corresponding to 740 proteins) and 3,816 (corresponding to 771 proteins) peptides were identified in DN and NDRD, respectively. Among these, the number of common peptides in both groups was 3,352 (corresponding to 720 proteins), whereas the number of proteins exclusive to DN and NDRD was 20 and 51, respectively (**Figures 5A, B**). Among the two groups of proteins identified, the relative abundance of 173 proteins between DN and NDRD changed (fold-change >2 or <0.5, p < 0.01) (**Figure 5C**), of which 160 proteins were significantly increased in NDRD and 13 in DN. Detailed information of the top 15 proteins with significant differences between DN and NDRD, including protein name, gene name, glycosylation site, and molecular weight information, is summarised in **Table 2**.

**Table 2:** Detailed information of the top 15 proteins with significant differences between the DN and NDRD groups.

Protein name	Gene	Glycosylation*^a^*	Mol. weight [kDa]	Fold change*^b^* (DN/NDRD)	p-Value*^c^*
Haptoglobin	HP	P^N,O^	45.2	0.09	<0.001
Complement C4-B	C4B; C4B_2; LOC100293534	P^N,O^	192.6	0.18	<0.001
Heparin cofactor 2	SERPIND1	P^N,O^	57	0.21	<0.001
Catalase	CAT	P^N,O^	59.7	0.35	<0.001
Complement C3	C3	P^N,O^	187	0.18	<0.001
Fibronectin	FN1	P^N,O^	262.5	0.13	<0.001
Alpha-2-macroglobulin	A2M	P^N,O^	163.2	0.15	<0.001
Triosephosphate isomerase	TPI1	P^N^	30.8	0.42	<0.001
Lactoylglutathione lyase	GLO1	P^O^	20.8	0.35	<0.001
Keratin, type I cytoskeletal 9	KRT9	P^N,O^	62	23.96	<0.001
Alpha-2-macroglobulin-like protein 1	A2ML1	P^N,O^	161	0.30	<0.001
Alpha-actinin-4	ACTN4	P^N,O^	104.8	0.16	<0.001
Leukotriene A-4 hydrolase	LTA4H	P^N,O^	69.2	0.20	<0.001
Inter-alpha-trypsin inhibitor heavy chain 4	ITIH4	P^N,O^	103.8	0.37	<0.001
Alpha-actinin-1	ACTN1	P^N,O^	103	0.27	<0.001

The protein expression level between NDRD and DN was compared and represented using the fold change.

DN, diabetic nephropathy; NDRD, non-diabetic renal disease.

^a^The potential N-linked and potential O-linked glycoproteins are analysed using software NetNGlyc 1.0 and NetOGlyc 4.0 Servers and shown as “P^N^” and “P°’; protein without typical glycosylation site is shown as “N”. ^b^The protein expression level between NDRD and DN was compared and represented using the fold change. The significant differences is setting with fold change > 2 or < 0.5 and p < 0.001.

^c^p-Value was calculated by two-tailed Student’s t-test.

### Bioinformatics Analysis of the Proteins Isolated From Diabetic Nephropathy and Non-Diabetic Renal Disease

**Figure 6:**
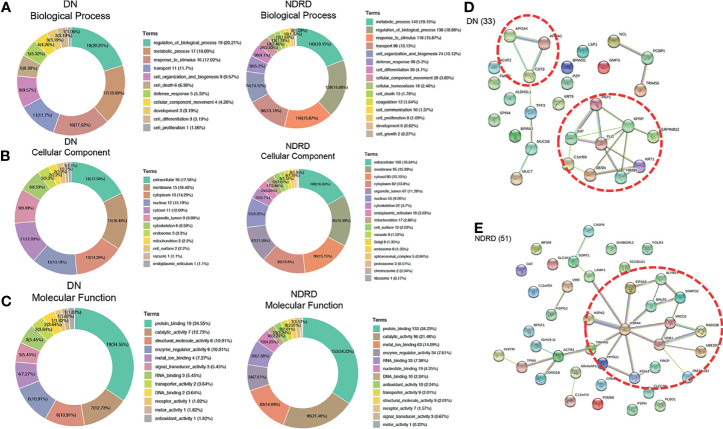
Bioinformatics analysis of differential glycoproteins isolated from DN and NDRD. Differential proteins were analysed using Gene Ontology (GO). DN, diabetic nephropathy; NDRD, non-diabetic renal disease. **(A)** Pie charts showing the biological processes of differential proteins between DN and NDRD. **(B)** Pie charts showing the cellular component of differential proteins between DN and NDRD. **(C)** Pie charts showing the molecular function of differential proteins between DN and NDRD. Next to their position are shown the associated term names on the chart. **(D)** STRING 9.0 was used to generalise and visualise the protein interaction network of differential proteins from DN. **(E)** STRING 9.0 was used to generalise and visualise the protein interaction network of differential proteins from NDRD. Line thickness represents the strength of the association between molecules. Networks with three or more protein interactions are shown. The confidence (score) required for protein association is high. The selected protein core complexes with important functions and proteins involved in the same biochemical reaction are marked with a red dotted line.

To better understand the biological functions of saliva glycoproteins that specifically bind to LEL lectin in DN, GO annotations and biological function of the isolated saliva proteins from the DN and NDRD groups were obtained using Blast2GO (http://www.blast2go.org/) software. That information was classified into cellular component, biological process, and molecular function. A total of 791 proteins were identified from DN and NDRD samples, and among these, 755 proteins were annotated successfully in biological process, cellular component, and molecular function (**Figure 5D**). As shown in **Figure 5D**, in the biological process chart, 506 proteins were involved in the metabolic process, and 502 proteins were involved in the regulation of biological processes. In terms of cellular component, 370 and 357 proteins were extracellular and membrane proteins, respectively, and 284 proteins were cytosolic proteins. In terms of molecular function, 538 proteins with binding ability accounted for the largest proportion, and those with a smaller proportion included 350 proteins with catalytic activity and 171 proteins with metal ion binding ability. A total of 20 and 51 proteins were specially identified in the DN and NDRD groups, respectively. Thirteen proteins were significantly upregulated in DN compared to NDRD (fold-change > 2, p < 0.01), and 160 proteins were significantly downregulated in NDRD compared to DN (fold-change > 2, p < 0.01). The potential differences in GO annotations and biological function between the two groups were analysed using pathway mapping and network analysis. As shown in **Figure 6A**, the differentially expressed proteins from DN and NDRD contributed to similar biological processes, such as metabolic processes and regulation of biological processes. However, several biological processes, including cellular homoeostasis, coagulation, and cell growth, were enriched in the NDRD group. In the cellular component charts, proteins related to Golgi, spliceosomal complex, proteasome, chromosome, and ribosome were only found in NDRD groups (**Figure 6B**). In the molecular function charts, the percentage of proteins with metal ion binding ability was lower in the DN group compared to the NDRD group (**Figure 6C**). Differential proteins of DN and NDRD (33 and 51, respectively) were used as defined in **Figures 6D, E**. The protein–protein interaction networks were unique in the identified proteins from the DN and NDRD groups. Two distinct protein–protein interaction sets were observed in the differential proteins of DN (**Figure 6D**), whereas one protein–protein interaction set was observed in the differential proteins of NDRD (**Figure 6E**). KEGG pathway analysis showed that the signal pathways enriched in the isolated proteins from the DN group included salivary secretion, PPAR signalling pathway, and extracellular matrix (ECM)–receptor interaction. In addition, the three most remarkable signalling pathways in the proteins from the NDRD group were complement and coagulation cascades, the pentose phosphate pathway, and the glycolysis/gluconeogenesis pathways (**Table S5**).

## Discussion

In recent years, diabetic kidney disease has attracted widespread attention (7, 8, 10). DN and NDRD differ in pathological characteristics, treatment response, and prognosis (37). Therefore, differentiating DN from NDRD has great clinical significance. Although renal biopsy is the gold diagnostic standard for distinguishing DN from NDRD, it is difficult to apply it to all patients because of its invasiveness and high technical proficiency required to perform the procedure. The detection of glycosylated salivary proteins by lectin has been studied in many disease fields because of its convenience, high efficiency, and accuracy.

To find an effective non-invasive diagnostic method, we used lectins to analyse the salivary glycopattern of DN and NDRD patients and to evaluate possible relationships with the severity and prognosis of DN patients. To verify the comparability between the training and validation cohorts, we compared 13 clinical indicators between the training and validation cohorts. There was no statistically significant difference in the clinical indicators between the two cohorts, which indicates that the model we built was not biased. Both models we established had their own advantages and high diagnostic accuracy for distinguishing DN from NDRD. The specificity of the logistic regression model was higher than that of the artificial neural network model in the validation cohort, whereas the sensitivity of the logistic regression model was lower than that of the artificial neural network model in the validation cohorts.

In addition, the correlation between glycopatterns and the severity and prognosis of DN was explored in this study. We analysed the correlation between the DN indicators and the 11 lectins that were differentially expressed in the DN and NDRD groups and explored whether they were related to the severity of DN. Notably, the levels of LEL, LCA, and VVA were observed to reflect the severity of DN, as revealed by Pearson’s correlation analysis. The eGFR was positively correlated with the level of LEL but negatively correlated with the level of LCA and VVA, whereas the Scr, classes of glomerular lesions, and scores of interstitial and vascular lesions were negatively correlated with the level of LEL but positively correlated with the level of LCA and VVA. The BUN was negatively correlated with the LEL level but positively correlated with the LCA level. The VVA level was positively associated with proteinuria. Knowing the severity of DN aids in judging the effects of treatment and choice of the treatment plan. Therefore, the discovery of non-invasive biomarkers that can reflect disease severity is of great clinical significance. We further performed Cox regression analysis on the five lectins that can reflect the severity of DN to determine whether they are related to the prognosis of DN. Surprisingly, two lectins were found to be related to the loss of kidney function and the time to start dialysis. Low levels of LEL and high levels of LCA have been demonstrated to accelerate the deterioration to ESRD. Therefore, the LCA and LEL levels can be used as non-invasive biomarkers to assess prognosis.

Salivary glycopatterns are good indicators of health status in many diseases (33). Abnormal glucose chain structures (N-glycans) are related to the occurrence and development of tumours (38–40). Disturbance of adhesion between cells and that between cells and the ECM leads to invasion and metastasis of tumour cells (41). It has been reported that outer-arm fucosylation and core-fucosylation detected by AAL and PSA in saliva were downregulated in gastric cancer patients compared with those in healthy individuals (42–44). In a study of pancreatic cancer cell lines, it was reported that the expression of *N*-acetylglucosaminyltransferase (GnT)-IVb was mainly downregulated in adjacent tissues, and the expression of GnT-IVb was mainly upregulated in tumour tissues (45). The deterioration of inflammatory and oxidative reactions in DN patients is related to the increased expression of abnormal glycation end-product receptors on the cell surface, which leads to aberrant intracellular signal transduction and ultimately worsens the disease (46, 47). The siaα2-6Gal/GalNAc glycoprotein, which was identified by SNA, has an increased abundance in the urine of DN patients (48) The Galb1-3GalNAc glycoprotein, which was identified by BPL and has a molecular weight of approximately 53 kDa, had a decreased abundance in the serum of DN patients compared with that of NDRD patients (49). Moreover, after 5 weeks of induction, the abundance of Gal/GalNAc glycan chain structure recognised by lectin PNA and RCA dramatically declined in DN mice compared with that of control mice (50).

LC-MS/MS analysis was used to separate salivary glycoproteins containing N-Acetyl-D-lactosamine (LacNAc) identified by LEL in both the DN and NDRD groups. Twenty proteins were found only in DN patients, and 51 proteins were identified only in NDRD patients. Altered glycosylation has been shown to be a characteristic of diabetes. Elevation of serum fucose levels was observed in diabetic rat and mouse models (51, 52). It was shown that the levels of α-1,6-fucosyltransferase and glycoproteins containing fucose residues were elevated in diabetic patients (52–54). There are significant differences in glycoproteins containing fuca1–2LacNAc, biantennary complex N-glycans, a-GalNAC, Galb1–3GalNAca-Thr/Ser, and LacNAc identified using UEAI, PHA-E, GSI, PNA, and RCA in kidney glycoprotein expression between rats with or without DN (50). A previous study by our group revealed that significant differences in serum glycoproteins containing Galb1-3GalNAc and terminal GalNAc identified by BPL occur between DN and NDRD patients, and the proteins were separated using LC-MS/MS (55). Differential protein analysis showed that the expression levels of keratin type I cytoskeletal 9 were significantly higher in the NDRD group, whereas the levels of haptoglobin were significantly lower in the NDRD group. Keratin type I cytoskeletal 9 consists of a cornified envelope and participates in cell death, cell organisation, and biogenesis *via* its activity as a structural molecule. Upregulated levels of haptoglobin affect neutrophil degranulation and scavenging of heme from the plasma. The signal networks of DN patients were associated with lipid metabolism. Apolipoprotein A4 (ApoA4), a member of the Apo family, is mainly found in enterocytes in the small intestine, with a smaller amount in the liver (56). Increased levels of ApoA4 are found in the serum from DM patients (57) owing to its association with hyperglycaemia and high-density lipoprotein levels (58). In addition, the development of DM and progression to DN show a significant relationship with the elevation of ApoA4 levels (59). Therefore, ApoA4 is a potential biomarker for predicting DN.

This study has the following advantages: first, compared with previous studies that used lectin microarrays to search for biomarkers after mixing samples, we tested each patient’s specimens individually on a lectin microarray for the first time. Therefore, our results are more accurate and reliable. Second, compared with previous studies that only used logistic regression to construct a diagnostic model for the results of the lectin microarray, the artificial neural network algorithm we used for the first time provided a non-invasive diagnostic model. Third, this study not only used differential lectin levels to establish a non-invasive diagnosis model for DN but also found their relationship with the severity and prognosis of DN.

This study has some limitations. First, this was a single-centre study, and multicentre and larger cohort studies are expected to further examine the diagnostic power of the model. Second, the role of the differentially expressed glycoproteins discovered in the pathogenesis of DN needs to be further explored. Third, it is advisable to extend the follow-up time to further confirm the relationship between lectin levels and the prognosis of DN.

In summary, our diagnostic models that were constructed by logistic regression and artificial neural networks could be used as non-invasive tools for distinguishing patients with DN and NDRD. The levels of AAL, LEL, LCA, VVA, and NPA could reflect DN severity, and the levels of LEL and LCA could reflect DN prognosis.

## Data Availability Statement

The original contributions presented in the study are publicly available. This data can be found here: ProteomeXchange, PXD030108.

## Ethics Statement

The studies involving human participants were reviewed and approved by the Ethics Committee of the Chinese PLA General Hospital (No. S2014-012-01). The patients/participants provided their written informed consent to participate in this study.

## Author Contributions

QH and HZ contributed to the study concept. QH and XW contributed to the manuscript draft and revision. QH, HY, JH, and ZT contributed to the data analysis. QH, XW, XD, and QL contributed to supervising subject enrolment and sample collection and data collection. QH, JW, and FY contributed to performing the experiment. HZ, GC, and DZ contributed to the review and editing. HZ verified the underlying data. All authors critically reviewed and edited the manuscript and consented to final publication. HZ had full access to all the data and had final responsibility for the decision to submit for publication.

## Funding

This research was funded by The National Natural Science Foundation of China (No. 61971441), the National Key R&D Program of China (Nos. 2021YFC1005300, 2018YFA0108803), and “Field Internal Science” Army Key Discipline Construction.

## Conflict of Interest

The authors declare that the research was conducted in the absence of any commercial or financial relationships that could be construed as a potential conflict of interest.

## Publisher’s Note

All claims expressed in this article are solely those of the authors and do not necessarily represent those of their affiliated organizations, or those of the publisher, the editors and the reviewers. Any product that may be evaluated in this article, or claim that may be made by its manufacturer, is not guaranteed or endorsed by the publisher.

## Abbreviations

DN, diabetic nephropathy; T2DM, type 2 diabetes mellitus; NDRD, non-diabetic renal disease; MN, membranous nephropathy; IgAN, IgA nephropathy; FSGS, focal segmental glomerulosclerosis; BCA, bicinchoninic acid; SDS-PAGE, sodium dodecyl sulphate–polyacrylamide gel electrophoresis; FA, formic acid; GO, Gene Ontology; NFI, normalised fluorescence intensity; HCA, hierarchical cluster analysis; PCA, principal component analysis; ROC, receiver operating characteristic; LCA, *Lens culinaris* lectin; VVA, *Vicia villosa* lectin; NPA, *Narcissus pseudonarcissus* lectin; ACA, *Amaranthus caudatus* lectin; PHA-E+L, *Phaseolus vulgaris* lectin; EEL, *Euonymus europaeus* lectin; AAL, *Aleuria aurantia* lectin; LTL, *Lotus tetragonolobus* lectin; LEL, *Lycopersicon esculentum* lectin; DBA, *Dolichos biflorus agglutinin* lectin; PWM, *Phytolacca americana* lectin; AUC, area under the curve; eGFR, estimated glomerular filtration rate; BUN, blood urea nitrogen; SCr, serum creatinine; ESRD, end-stage renal disease; HR, hazard ratio; GnT-IVb, *N*-acetylglucosaminyltransferase-IVb; LacNAc, N-Acetyl-D-lactosamine; ApoA4, apolipoprotein A4.
