# Supplementary material for: Salivary Glycopatterns as Potential Non-Invasive Biomarkers for Diagnosing and Reflecting Severity and Prognosis of Diabetic Nephropathy
Source: Front Endocrinol (Lausanne). 2022 Mar 31;13:790586. doi: 10.3389/fendo.2022.790586 (PMC9009518; doi:10.3389/fendo.2022.790586)
Supplement: Supplementary file 1 [file DataSheet_1.docx]

**Table S1. Baseline characteristics of patients in training cohort and validation cohort.**

|  | Training cohort | Validation cohort | P |
| --- | --- | --- | --- |
| Male % | 76.19% | 76.36% | 0.980 |
| Age (years) | 52.357±10.430 | 52.764±10.013 | 0.807 |
| Creatinine ( μmol/L) | 142.334±70.735 | 142.964±68.640 | 0.956 |
| eGFR (ml/min/1.73m2) | 58.178±27.877 | 56.817± 26.446 | 0.759 |
| SBP (mmHg) | 146.333±21.684 | 146.073±23.270 | 0.942 |
| DBP (mmHg) | 86.683±12.353 | 86.145±12.670 | 0.790 |
| BMI (kg/cm2) | 26.216±3.816 | 26.937±3.640 | 0.238 |
| Urinary protein (g/24 h) | 4.333±4.515 | 4.452±3.219 | 0.841 |
| HGB（g/L） | 121.444±20.489 | 120.927±23.890 | 0.889 |
| Serum ALB（g/L） | 33.637±8.265 | 31.329±8.101 | 0.084 |
| FBG (mmol/l) | 6.652±2.444 | 6.190±2.428 | 0.243 |
| Urea（mmol/L） | 8.651±4.218 | 8.497±5.202 | 0.834 |
| SUA (umol/L) | 377.286±99.158 | 378.582±85.306 | 0.929 |

Notes: DN, diabetic nephropathy; NDRD, non-diabetic renal disease; eGFR, estimated glomerular filtration rate; SBP, systolic blood pressure; DBP, diastolic blood pressure; BMI, body mass index; HGB, hemoglobin; ALB, albumin; FBG, fasting blood-glucose; UA, serum uric acid. The estimated glomerular filtration rate calculated with the Chronic Kidney Disease Epidemiology Collaboration (CKD-EPI) equation.

**Table S2. Diabetes related clinical information of the DN and NDRD groups.**

|  | DN group | NDRD group | P |
| --- | --- | --- | --- |
| FBG (mmol/l) | 7.01±2.89 | 6.94±0.96 | 0.639 |
| HbA1c(%) | 7.03±1.34 | 6.61±3.73 | 0.425 |
| DM duration (years) | 8.87±4.06 | 8.49±3.91 | 0.524 |
| Take hypoglycemic drugs regularly | 89.90% | 91.46% | 0.719 |

Notes: DN, diabetic nephropathy; NDRD, non-diabetic renal disease; FBG, fasting blood-glucose; HbA1c, hemoglobin A1c; DM, diabetes mellitus.

**Table S3. Detailed information regarding the ROC analysis of the constructive models in the training cohort and validation cohort with logistic regression and artificial neural network analysis, respectively.**

|  | Sensitivity | Specificity | Accuracy | AUC |  |
| --- | --- | --- | --- | --- | --- |
| **Logistic Regression Analysis** | | | | | |
| **Training Cohort** | 0.700 | 0.946 | 0.810 | 0.892 |  |
| **Validation Cohort** | 0.690 | 0.923 | 0.800 | 0.867 |  |
| **Artificial Neural Network Analysis** | | | | | |
| **Training Cohort** | 1.000 | 1.000 | 1.000 | 1.000 |  |
| **Validation Cohort** | 0.862 | 0.769 | 0.818 | 0.879 |  |

Notes: ROC, receiver operating characteristic; AUC, the area under the ROC curve.

**Table S4. Multivariate Cox regression analysis for dialysis-free survival of patients with diabetic nephropathy.**

| Items | β | SE | Wald | P | HR | 95% CI | |
| --- | --- | --- | --- | --- | --- | --- | --- |
|  |  |  |  |  |  | **Lower** | **Upper** |
| **AAL** | 1.380 | 6.126 | 0.051 | 0.820 | 3.975 | < 0.001 | 651712.093 |
| **LEL** | -69.206 | 19.255 | 12.918 | < 0.001 | < 0.001 | < 0.001 | < 0.001 |
| **LCA** | 13.501 | 4.936 | 7.482 | 0.006 | 730046.848 | 45.927 | 11604701716.844 |
| **VVA** | 0.978 | 6.784 | 0.021 | 0.885 | 2.658 | < 0.001 | 1583391.749 |
| **NPA** | -13.664 | 12.293 | 1.236 | 0.266 | < 0.001 | < 0.001 | 33840.517 |

Notes: β, regression coefficient; SE, standard error; HR, hazard ratio; CI, confidence interval. *p<0.05; **p<0.01; and ***p<0.001.

**Table S5. The top 3 signaling pathways with enrichment degree are analyzed by KEGG**.

|  | Count | P Value | Fold Enrichment |
| --- | --- | --- | --- |
| DN |  |  |  |
| Salivary secretion | 7 | < 0.001 | 13.657 |
| PPAR signaling pathway | 3 | 0.037 | 7.514 |
| ECM-receptor interaction | 3 | 0.040 | 5.786 |
| NDRD |  |  |  |
| Complement and coagulation cascades | 21 | < 0.001 | 9.024 |
| Pentose phosphate pathway | 11 | < 0.001 | 11.247 |
| Glycolysis / Gluconeogenesis | 14 | < 0.071 | 6.196 |

Notes: DN, diabetic nephropathy; NDRD, non-diabetic renal disease; PPAR, peroxisome proliferators-activated receptors; ECM, extracellular matrix;
